# Supplementary material for: Live-Cell Cardiac-Specific High-Throughput Screening Platform for Drug-Like Molecules That Enhance Ca2+ Transport
Source: Cells. 2020 May 8;9(5):1170. doi: 10.3390/cells9051170 (PMC7291019; doi:10.3390/cells9051170)
Supplement: Supplementary file 1 [file cells-09-01170-s001.pdf]

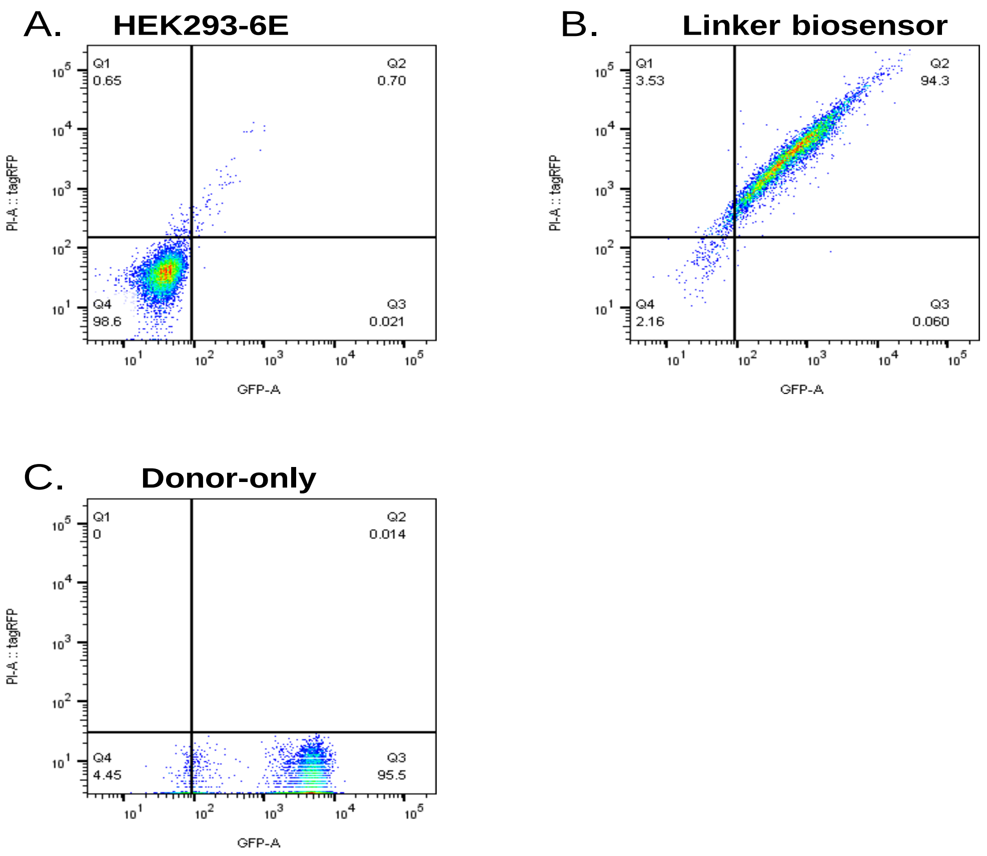

Figure S1 Flow cytometry 2D scatter plots showing GFP and RFP expression from control cell lines **A.** Untransfected parental cell line HEK293-6E **B.** Stable HEK293-6Ecell line expressing linker biosensor shows equal signals from GFP and RFP (Q2) **C.** Stable HEK293-6e cell line expressing the donor-only control corresponding to the fusion biosensor.

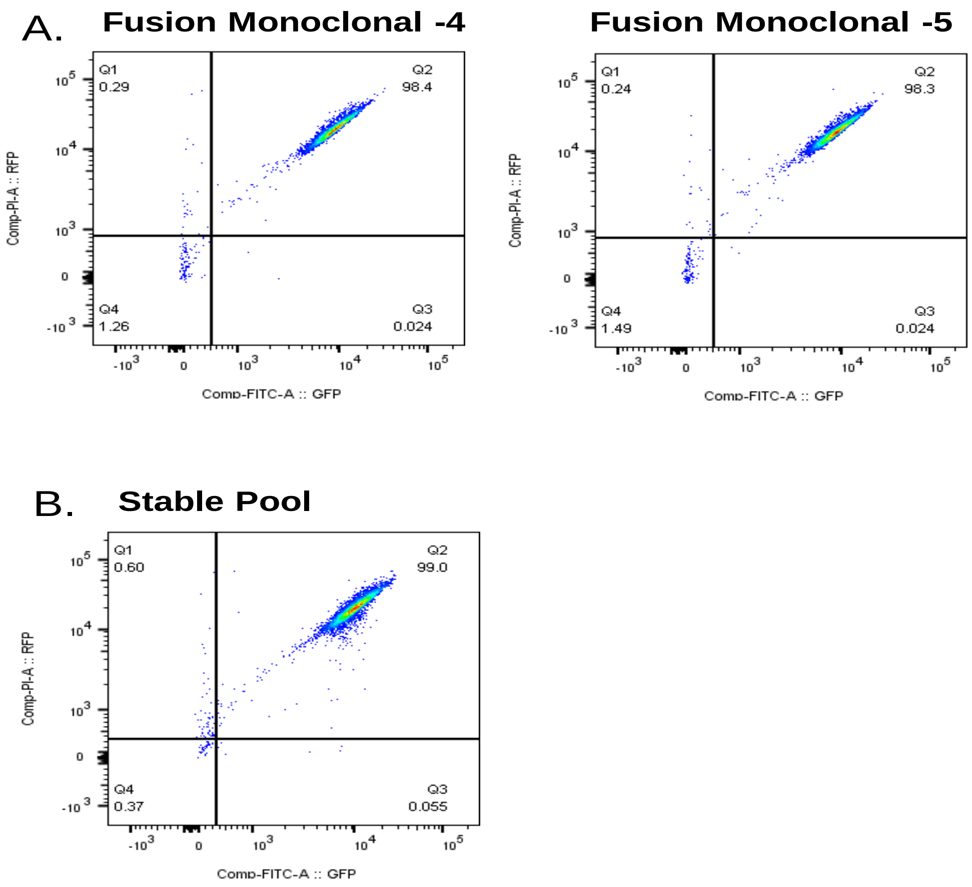

Figure S2 Flow cytometry 2D scatter plots of GFP and RFP expression. **A.** Two fusion biosensor monoclonal stable cell lines generated by serial dilution from the stable pool generated after antibiotic selection and FACS isolation. **B.** Source of monoclonal cell lines shown in panel A is a stable pool of fusion biosensor expressing cells, which also shows high GFP and RFP expression.

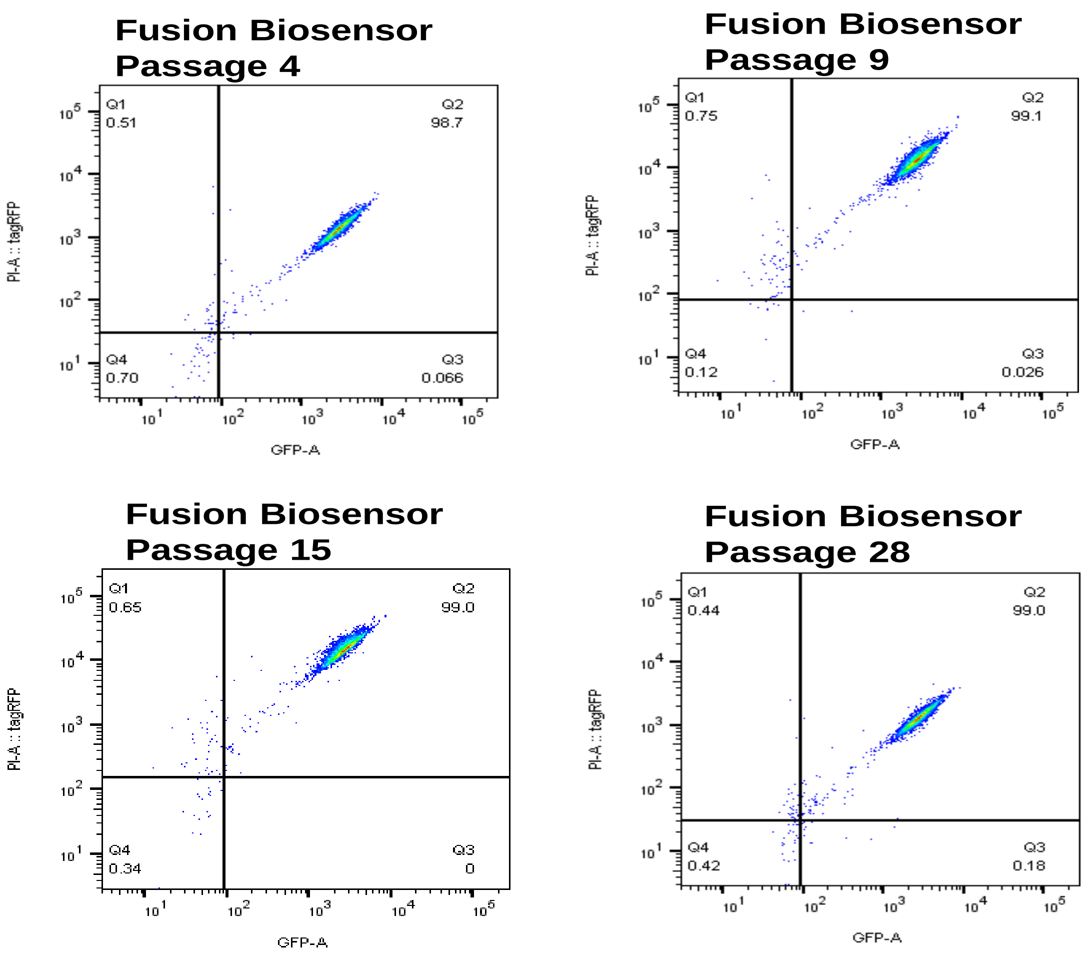

Figure S3 Flow Cytometry 2D scatter plots of GFP and RFP expression of the stable cell line expressing the fusion biosensor shown over multiple passages in cell culture. GFP and RFP expressing remains stable over multiple passages.

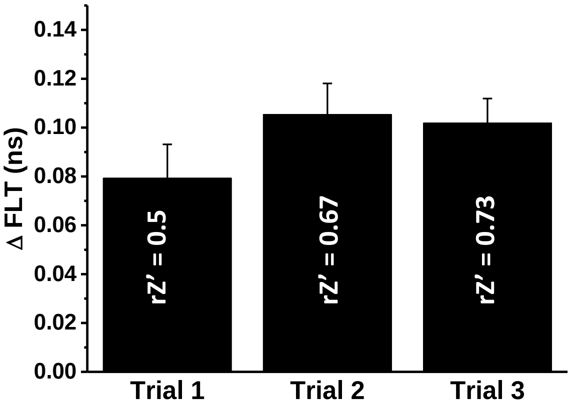

Figure S4 HTS assays quality parameter (rZ') calculated from ΔFLT of the known SERCA inhibitor, thapsigargin. Three separate trials show a large FLT change from a 200 nM saturating dose of thapsigargin compared to DMSO control wells. Based on this FLT change, the calculated rZ′ was ≥0.5 for all three trials. N=192 wells for each condition.

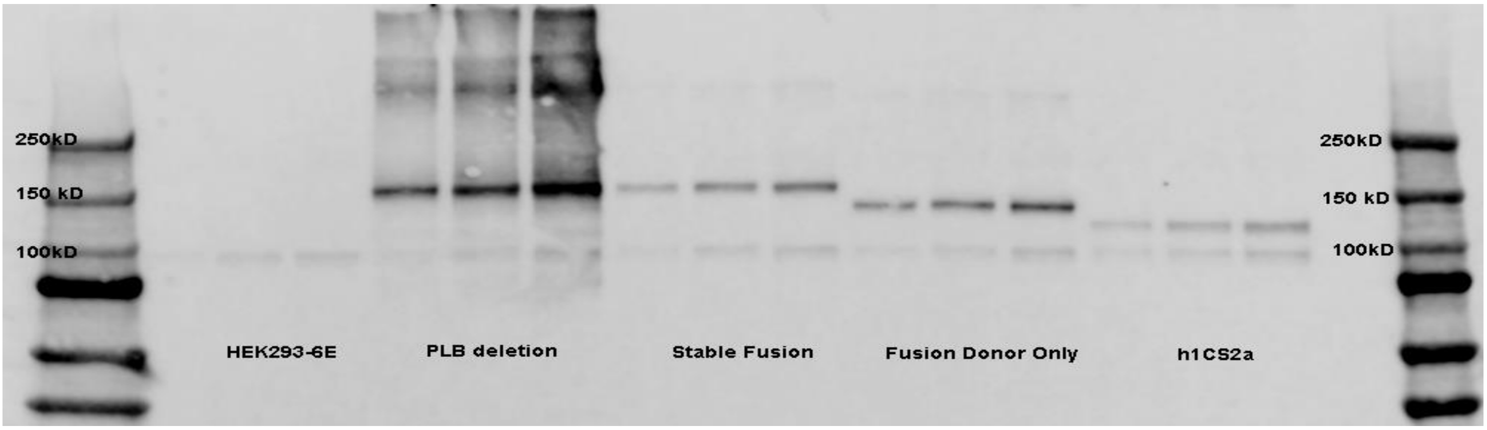

Figure S5 Full western blot corresponding to Figure 3A. Cell lines shown are: untransfected HEK293 cells, fusion biosensor construct with PLB deletion, fusion biosensor, donor-only control with GFP fused to PLB, and a one-color SERCA2a control cell line with GFP tagged at the N-terminus as a control for ATPase activity assays.

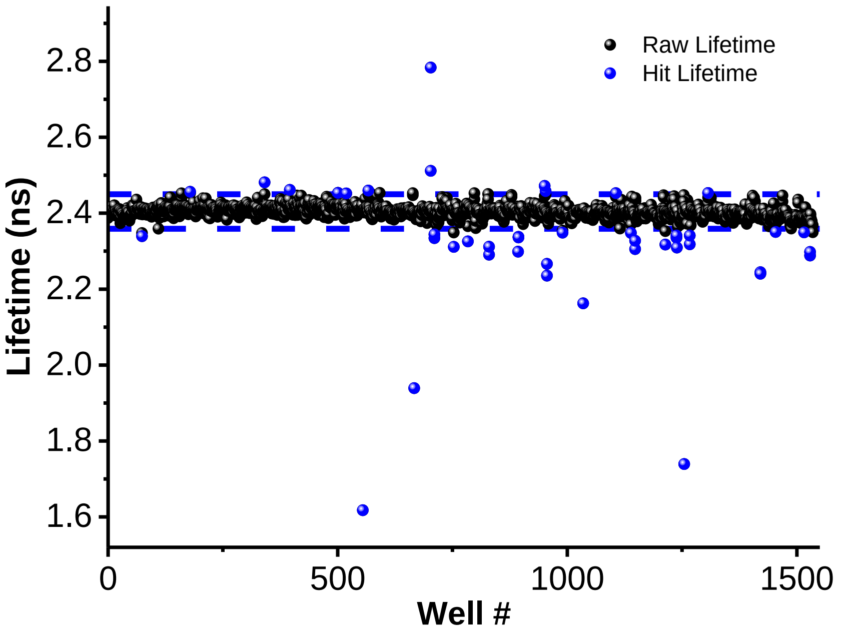

Figure S6 FUSION LOPAC screen data showing the raw lifetime. Compounds that change the SERCA-PLB fusion biosensor FLT by ≥5SD were selected as Hits (blue). FCs were flagged and excluded from Hits. Hits selected as being 5SD from the DMSO control wells are shown in blue.
